# E1A enhances cellular sensitivity to DNA-damage-induced apoptosis through PIDD-dependent caspase-2 activation

**DOI:** 10.1038/cddiscovery.2016.76

**Published:** 2016-10-31

**Authors:** Jay R Radke, Zeba K Siddiqui, Iris Figueroa, James L Cook

**Affiliations:** 1 Research Section, Edward Hines, Jr. VA Hospital, 5000 S 5th Ave., Hines, IL 60141, USA; 2 Division of Infectious Diseases, Loyola University Medical Center; Infectious Diseases and Immunology Research Institute, Loyola University Chicago—Stritch School of Medicine, Maywood, IL, USA; 3 Department of Microbiology and Immunology, Loyola University Chicago—Stritch School of Medicine, Maywood, IL, USA; 4 Department of Medicine, Section of Infectious Diseases, University of Illinois at Chicago, Chicago, IL, USA

## Abstract

Expression of the adenoviral protein, E1A, sensitizes mammalian cells to a wide variety of apoptosis-inducing agents through multiple cellular pathways. For example, E1A sensitizes cells to apoptosis induced by TNF-superfamily members by inhibiting NF-kappa B (NF-*κ*B)-dependent gene expression. In contrast, E1A sensitization to nitric oxide, an inducer of the intrinsic apoptotic pathway, is not dependent upon repression of NF-*κ*B-dependent transcription but rather is dependent upon caspase-2 activation. The latter observation suggested that E1A-induced enhancement of caspase-2 activation might be a critical factor in cellular sensitization to other intrinsic apoptosis pathway-inducing agents. Etoposide and gemcitabine are two DNA damaging agents that induce intrinsic apoptosis. Here we report that E1A-induced sensitization to both of these agents, like NO, is independent of NF-*κ*B activation but dependent on caspase-2 activation. The results show that caspase-2 is a key mitochondrial-injuring caspase during etoposide and gemcitabine-induced apoptosis of E1A-positive cells, and that caspase-2 is required for induction of caspase-3 activity by both chemotherapeutic agents. Expression of PIDD was required for caspase-2 activation, mitochondrial injury and enhanced apoptotic cell death. Furthermore, E1A-enhanced sensitivity to injury-induced apoptosis required PIDD cleavage to PIDD-CC. These results define the PIDD/caspase-2 pathway as a key apical, mitochondrial-injuring mechanism in E1A-induced sensitivity of mammalian cells to chemotherapeutic agents.

## Introduction

Products of the adenoviral early region 1A gene (E1A) are critical for efficient adenoviral replication.^1^ E1A is a modulator of cellular and viral gene transcription and the primary mediator of cell cycle induction during quiescent cell infection. As a result of these cell cycle effects, E1A can immortalize cells during either abortive infection or stable expression, similarly to other DNA tumor viral and cellular oncogenes.^2,3^ As an apparent side effect of its cell cycle regulatory activity, E1A expression increases the sensitivity of cells to a variety of potentially proapoptotic stimuli, including immunological and physical injuries and chemotherapeutic agents.^4^ The cellular pathways and molecular mechanisms through which E1A induces cellular sensitivity to apoptosis triggered by these diverse stimuli remains incompletely defined.^5–18^ One possibility is that E1A alters a common cellular regulatory pathway, resulting in increased sensitivity to proapoptotic stimuli. A potential candidate is the cellular transcription factor, NF-*κ*B.

NF-*κ*B regulates expression of many antiapoptotic proteins and has therefore been termed the central cellular regulator of apoptosis.^19^ Its role in preventing apoptosis induced by TNF-superfamily members is well established. It has been proposed that NF-*κ*B responses are required to prevent chemotherapeutic and irradiation induced apoptosis.^20,21^ However, the role of NF-*κ*B in preventing apoptosis induced by chemotherapeutic agents is controversial. We and others have established that E1A-induced cellular sensitivity to apoptosis induced by TNF-superfamily members is mediated by inhibition of stimulus-induced, NF-*κ*B-dependent transcription.^6,7,18^

The activated macrophage, one component of the innate immune antitumor defense, induces cytolysis of E1A-expressing tumor cells through multiple mechanisms. Activated macrophage-produced nitric oxide (NO) is the major cytolytic mediator.^9^ We have reported that NO induces apoptosis of E1A-expressing cells through a caspase-2-dependent pathway, independently of cellular NF-*κ*B responses.^22^ Furthermore, we identified caspase-2 as an upstream initiator of mitochondrial injury of NO-treated, E1A-expressing cells undergoing apoptosis.^22^ These results suggested that E1A might sensitize cells through the intrinsic apoptotic pathway, independently of cellular NF-*κ*B responses.

Caspase-2 is the most conserved member of the caspase family. Caspase-2 activation has been linked to cell death associated with various cellular injuries that cause metabolic imbalance, DNA damage, ER stress or mitotic distress.^23^ Caspase-2 contains a caspase activation and recruitment domain (CARD), through which it is recruited to high molecular weight complexes for activation, similarly to initiator caspases-1, -8 and -9. The caspase-2 PIDDosome has been implicated as one such high molecular weight activation complex.

The PIDDosome is a high molecular weight complex composed of procaspase-2, p53-induced protein with a death domain (PIDD) and RIP (receptor interacting protein)-associated death domain containing protein (RAIDD).^24–26^ To activate procaspase-2, PIDD must undergo two sequential cleavage events that first yield PIDD-N and PIDD-C and then yield PIDD-CC as a cleavage product of PIDD-C.^27^ Processing to PIDD-CC, through cleavage of PIDD-C at serine 588 (S588), is required for caspase-2 activation. Mutation of S588 to alanine prevents the processing of PIDD-C to PIDD-CC and prevents PIDDosome activation of caspase-2.^27^ PIDD has been shown to be required for apoptosis induced by DNA damaging agents.^27–29^ However, the role of PIDD in caspase-2 activation is not universal.^30–32^

In the present studies, we investigated the role of capase-2 in E1A-mediated cellular sensitivity to intrinsic apoptosis induced by two DNA damaging agents, etoposide and gemcitabine. Our data show that E1A sensitizes cells to apoptosis through a mechanism that is dependent upon enhanced caspase-2 activation but independent of injury-induced NF-*κ*B activation. The results indicate that the E1A-enhanced caspase-2 effect is upstream of mitochondrial injury, suggesting caspase-2 is an apical caspase activated in response to intrinsic injury in E1A-expressing cells. In addition, enhanced activation of casapse-2 required cleavage of PIDD to PIDD-CC. These results indicate that E1A expression renders cells sensitive to intrinsic apoptosis-inducing agents as a result of E1A-related increased caspase-2 activation through the PIDDosome.

## Results

### Sensitivity of E1A-positive human and mouse cells to apoptosis induced by DNA damaging chemotherapeutic agents

**Figure 1:**
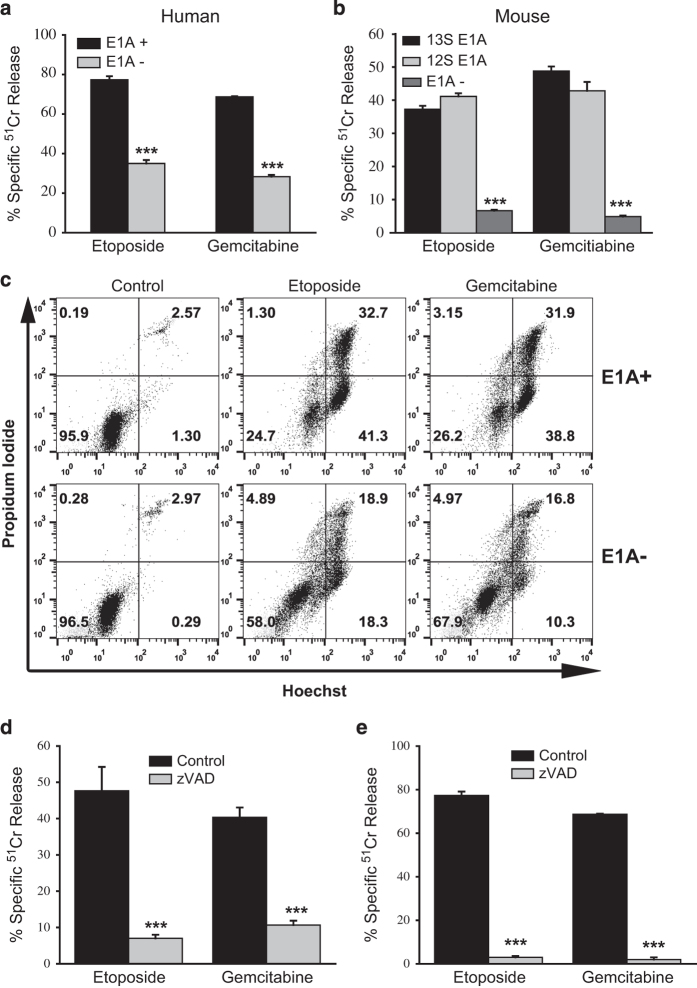
Characterization of the cytotoxic sensitivity of E1A-expressing mouse and human cells to etoposide and gemcitabine. (**a**) Genomic E1A-expressing (E1A+) or E1A-negative (E1A−) human H4 fibrosarcoma cells were treated with etoposide (1 *μ*M) or gemcitabine (1.5 *μ*M) for 18 h, and specific Cr^51^ release was determined (mean±S.E.M.; *n*=3, ***P<0.001). (**b**) E1A 13S-positive (13S E1A), E1A 12S-positive (12S E1A) and E1A-negative (E1A−) NIH-3T3 mouse cells were treated with etoposide (400 *μ*M) or gemcitabine (50 *μ*M) for 18 h, and specific Cr^51^ release was determined (mean±S.E.M.; *n*=3, ***P<0.001). (**c**) Hoechst/PI staining patterns of E1A 12S-positive (E1A+) or E1A-negative (E1A−) mouse NIH-3T3 cells following treatment with etoposide (400 *μ*M) or gemcitabine (50 *μ*M) for 18 h. Numbers in the quadrants denote the percentages of cells present in each quadrant out of the total cell population analyzed. Data are representative of four independent experiments. (**d** and **e**) Mouse (**d**) E1A 12S-positive or human (**e**) genomic E1A-positive cells were treated with etoposide or gemcitabine as above in the absence (black bars) or presence (gray bars) of the pan-caspase inhibitor, zVAD (100 *μ*M), for 18 h, and the specific Cr^51^ release was determined (mean±S.E.M.; *n*=3, ***P<0.001).

E1A expression sensitizes mammalian cells to DNA damaging agents that induce apoptosis through the intrinsic pathway.^33^ We used two chemotherapeutic agents that induce DNA damage by distinct mechanisms – etoposide, a topoisomerase II inhibitor that causes both single- and double-stranded DNA breaks, and gemcitabine, a nucleoside analog that causes DNA chain termination. E1A-expressing human and mouse fibroblastic cell lines were tested for sensitivity to these two agents (Figure 1).^11,34^ Expression of genomic E1A (Figure 1a, human cells) or cDNAs of either major E1A splice variant, 13S or 12S (Figure 1b, mouse cells), induced increased sensitivity to cytotoxicity by both chemotherapeutic agents. The apoptotic nature of the cell death responses was confirmed by flow cytometry (Figure 1c), which revealed an increased proportion of treated, E1A-positive cells in the Hoechst-positive/propidium iodide-negative fraction (right lower quadrant), when compared with treated, E1A-negative cells. Treated, E1A-expressing mouse and human cells exhibited identical, differential staining patterns in these flow cytometry studies. Apoptosis induced in E1A-positive cells by both chemotherapeutic agents was caspase-dependent, as evidenced by the blockade of cell death with the pan-caspase inhibitor, zVAD (Figures 1d and e).

### NF-*κ*B independence of E1A-induced chemosensitivity

**Figure 2:**
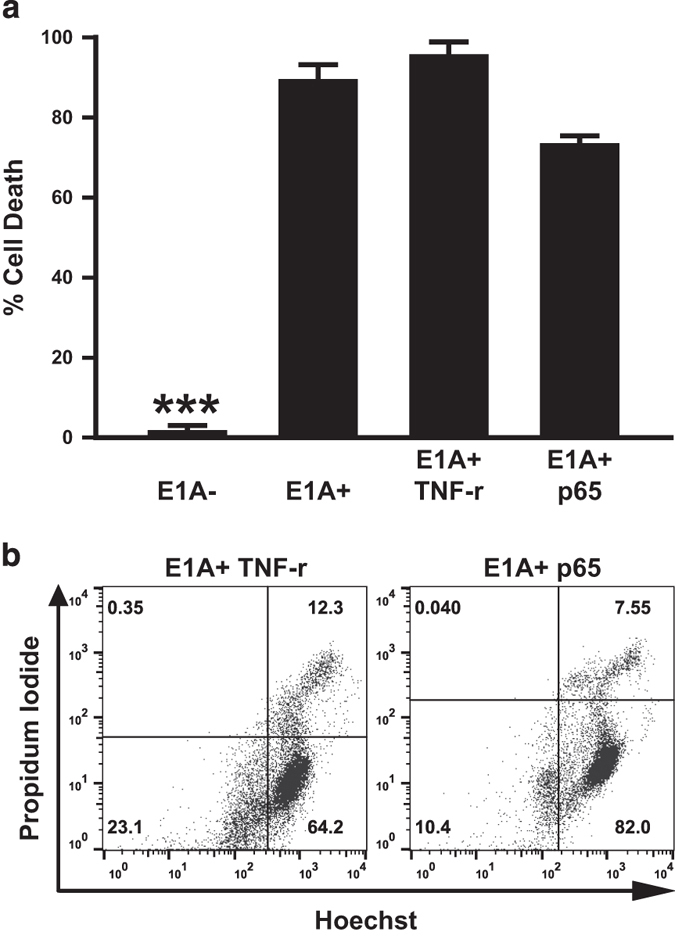
Role of NF-*κ*B cellular responses in etoposide-induced apoptosis. (**a**) Four different mouse cell lines – E1A− controls; E1A+ controls; TNF-resistant E1A+ cells (E1A+ TNFr); or p65/RelA-overexpressing E1A+ cells (E1A+ p65) cells – were treated with 400 *μ*M etoposide for 18 h. Cell viability was determined by MTS staining and expressed as % cell death (mean±S.E.M.; *n*=3, ***P<0.001). (**b**) Hoechst/PI staining patterns of E1A+ TNFr or E1A+ p65 cells following treatment with etoposide (400 *μ*M) for 18 h.

Repression of NF-*κ*B-dependent cellular responses can sensitize cells to stimuli of the intrinsic apoptotic pathway.^35–37^ However, we have observed that E1A sensitizes cells to apoptosis caused by NO, an inducer of the intrinsic apoptosis pathway, through a mechanism that is independent of NF-*κ*B activity.^22^ Based on those results, we postulated that the mechanism(s) through which E1A sensitizes cells to other intrinsic apoptosis pathway stimuli, such as the chemotherapeutic agents being studied here, would also be NF-*κ*B-independent. E1A-positive mouse cells that were either selected for NF-*κ*B-dependent resistance to TNF*α* or made TNF resistant by overexpression of NF-*κ*B p65/RelA, but that remained sensitive to NO-induced apoptosis, were tested for sensitivity to etoposide-induced apoptosis (Figure 2).^22^ TNF-resistant (E1A+ TNFr) or p65/RelA-overexpressing (E1A+ p65) E1A-positive cells showed no significant reduction in sensitivity to etoposide-induced cell death compared with the E1A-positive control cells (Figure 2a). Furthermore, both TNF-resistant and p65/RelA-overexpressing E1A-positive cells underwent apoptotic cell death identical to that observed with E1A-positive control cells (compare Figure 2b with Figure 1c). These results are similar to those observed with NO-induced apoptosis of E1A-positive cells, suggesting that E1A might use a common mechanism to induce sensitivity to these two drug-induced intrinsic apoptotic injuries that is independent of NF-*κ*B-dependent cellular responses.^22^

### E1A sensitizes cells to DNA damaged induced apoptosis through a caspase-2 dependent pathway

**Figure 3:**
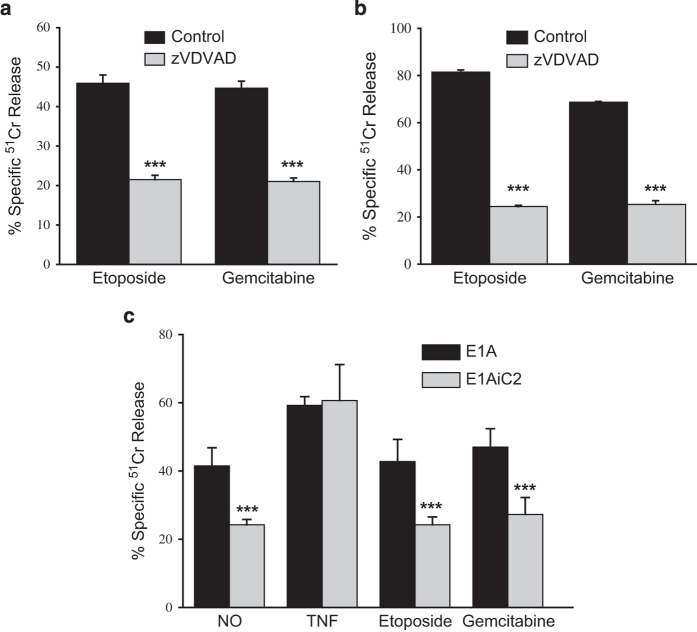
Role of caspase-2 in apoptosis induced by etoposide or gemcitabine. Mouse cells expressing E1A 12S (**a**) or human cells expressing genomic E1A (**b**) were treated with etoposide or gemcitabine in the absence (black bars) or presence (gray bars) of the caspase-2-specific inhibitor, zVDVAD (200 *μ*M) for 18 h, and specific Cr^51^ release was determined (mean±S.E.M.; *n*=3, ***P<0.001). (**c**) E1A 12S-positive mouse cells (E1A) or caspase-2 siRNA-expressing E1A 12S-positive mouse cells (E1AiC2) were treated with the chemical NO generator, DETANoNoate (NO) (250 *μ*M), TNF*α* (20 ng/ml), etoposide (400 *μ*M) or gemcitabine (50 *μ*M) for 18 h, and specific Cr^51^ release was determined (mean±S.E.M.; *n*=3, ***P<0.001).

We have reported that E1A sensitizes cells to NO through a caspase-2-dependent mechanism.^22^ It has been suggested that apoptosis induced by DNA damaging agents, such as etoposide, also depends on caspase-2 activation.^38^ We postulated that apoptosis of E1A-positive cells induced by etoposide and gemcitabine would also depend on caspase-2 activation. To test this hypothesis, E1A-positive mouse and human fibroblasts were treated with etoposide or gemcitabine in the presence or absence of the caspase-2-selective inhibitor, zVDVAD-fmk. The results in Figures 3a and b showed that inhibition of caspase-2 activity with zVDVAD decreased cell death of both mouse and human E1A-positive fibroblasts treated with etoposide or gemcitabine. To validate the inhibitor studies, we tested an E1A-positive caspase-2 shRNA mouse cell line (E1A-iC2) that is resistant to NO-induced cell death but sensitive to TNF-induced cell death.^22^ There was a significant reduction in the sensitivity of the caspase-2 shRNA-expressing E1A-positive cells to both etoposide and gemcitabine compared with E1A-positive control cells (Figure 3c). The pattern of caspase-2 expression dependence of cytotoxic sensitivity was similar to that observed with NO injury and contrasted with the lack of correlation between caspase-2 expression dependence observed with TNF injury.

### Caspase-2 is an apical mitochondrial-injuring caspase

**Figure 4:**
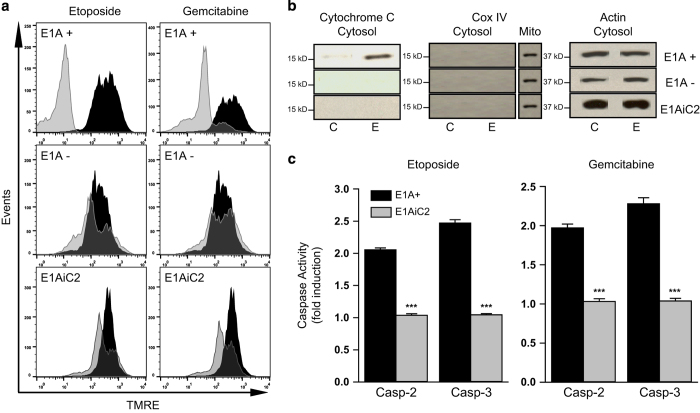
Caspase-2-mediated mitochondrial injury and effector caspase activation in mouse cells. (**a**) TMRE staining of E1A 12S-positive cells (E1A+), E1A-negative cells (E1A−) or caspase-2 siRNA expressing E1A 12S-positive cells (E1AiC2) following treatment with etoposide (400 *μ*M) or gemcitabine (50 *μ*M) for 18 h. Treated cell results (gray histogram) are overlayed on those for untreated cells (black histogram). Data presented are representative of three independent experiments. (**b**) Mitochondrial release of cytochrome *c* into the cytosol in E1A 12S-positive (E1A+), E1A-negative (E1A−) or caspase-2 siRNA-expressing E1A 12S-positive (E1AiC2) cells, following treatment with etoposide (400 *μ*M) for 4 h (E) or untreated control (C). Data presented are representative of three independent experiments. (**c**) Caspase-2 and caspase-3 activity in E1A 12S-positive (E1A+) or caspase-2 siRNA expressing E1A 12S-positive (E1AiC2) cells following treatment with etoposide (400 *μ*M) or gemcitabine (50 *μ*M) for 4 h. Activity is expressed as fold induction compared with untreated cells (1.0) (mean±S.E.M.; *n*=3, ***P<0.001).

Recent reports suggest that caspase-2 is an apical (initiator) caspase.^39–41^ We observed that caspase-2 is involved in rapid injury of mitochondria following exposure to NO (unpublished data). Therefore, we examined whether etoposide and gemcitabine treatment of E1A-positive cells resulted in caspase-2-dependent mitochondrial injury. We assayed mitochondrial membrane potential (MMP) following 18 h of cellular exposure to etoposide or gemcitabine by staining with mitochondria-specific TMRE. Mitochondria with normal MMP fluoresce as a result of TMRE accumulation, whereas reduced MMP causes decreased TMRE-related fluorescence (with a shift to the left on the histogram). E1A-positive cells showed a loss of MMP following exposure to both chemotherapeutic agents compared with untreated E1A-positive cells (Figure 4a, top panel, gray/treated *versus* black/untreated cells). Conversely, E1A-negative cells retained MMP fluorescence following treatment, with little or no shift to the left, compared with untreated control cells (Figure 4a, middle panel). To test the role of caspase-2 activation in the loss of MMP following chemotherapeutic drug exposure, we measured TMRE staining of the E1A-iC2 (caspase-2 shRNA expressing) cells following drug treatment. These cells retained MMP, despite drug injury (Figure 4a, bottom panel). These results indicated that caspase-2 expression is required for etoposide and gemcitabine induced injury of mitochondria in E1A-positive cells, in a manner similar to that observed with NO-induced apoptosis.^22^

Treatment of E1A-positive control cells with etoposide or gemcitabine resulted in cytochrome *c* release from mitochondria into the cytosol, whereas no such cytochrome *c* release was noted with E1A-negative or E1A-iC2 cells (Figure 4b, control (C) *versus* etoposide treated (E)). Antibody to Cox IV, a mitochondrial marker, was used to validate the quality of separation of mitochondria from the cytosol. As observed with the loss of MMP, caspase-2 expression in E1A-positive cells was required for drug-induced mitochondrial release of cytochrome *c*.

There are reports that caspase-2 can be activated by caspase-3.^42^ To test this, we measured caspase-3 activity in E1A-positive, E1A-negative and E1A-positive caspase-2-negative (E1A-iC2) cells (Figure 4c). Treatment of E1A-positive cells with either etoposide or gemcitabine activated caspase-3. However, this drug-induced caspase-3 activation was blocked when caspase-2 expression was blocked by shRNA (E1A-iC2) (gray bars). These data, along with those on MMP and cytochrome *c* release, indicated that drug-induced caspase-2 activation occurred upstream of mitochondrial injury and subsequent caspase-3 activation, thus placing caspase-2 as an apical, mitochondria-injuring caspase in the context of chemotherapeutic drug-induced apoptosis of E1A-positive cells.

### PIDD is required for caspase-2-dependent apoptosis and loss of MMP in E1A-positive cells

**Figure 5:**
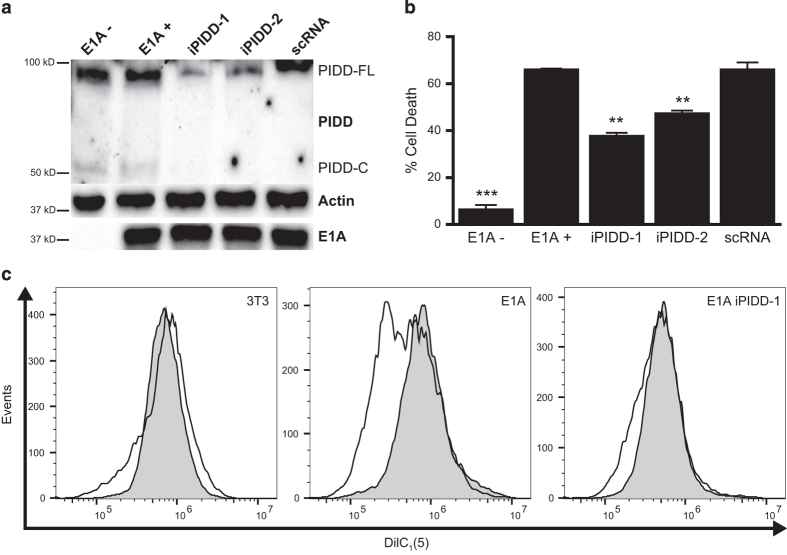
Requirement for PIDD in E1A-enhanced mouse cell sensitivity to etoposide. (**a**) Western blot for the expression of mouse PIDD, actin and E1A in E1A-negative (E1A−), E1A 12S-positive (E1A+), PIDD shRNA expressing E1A+ cells (iPIDD-1 and iPIDD-2) and E1A+ cells expressing scrambled control shRNA (scRNA). Full-length PIDD (PIDD-FL) and PIDD-C are indicated. (**b**) E1A−, E1A+, E1A+ iPIDD and E1A+ scRNA cells were treated with etoposide for 18 h. Cell viability was determined by MTS staining and expressed as % cell death (mean±S.E.M.; *n*=3; ***P<0.001, **P=0.003 compared with E1A+ controls). (**c**) DilC_1_ (5) staining of E1A−, E1A+ and E1A+ iPIDD-1 cells, following treatment with etoposide (400 *μ*M) for 18 h. Histograms for etoposide-treated cells (unshaded histograms) are overlayed on those for untreated cells (gray histograms). Data presented are representative of three independent experiments.

PIDD has been implicated in the p53-mediated death response of cells to certain proapoptotic agents, such as the DNA damaging chemotherapeutic drugs used in these studies.^27,43^ Furthermore, we have reported that E1A-induced sensitization of mouse fibroblasts to etoposide is strictly p53-dependent.^17^ Lentiviruses expressing GFP and either shRNA against mouse PIDD or scrambled shRNA (scRNA) were used to infect E1A-positive mouse cells. Cell clones were selected in puromycin and screened for GFP by FACS. High GFP expressing cells were screened for PIDD, actin and E1A expression (Figure 5a). Two shRNA PIDD lines, E1A-iPIDD-1 (iPIDD-1) and E1A-iPIDD-2 (iPIDD-2), had a marked decrease in PIDD expression, while maintaining E1A expression levels similar to uninfected E1A-positive cells and E1A-positive cells expressing scRNA. iPIDD-1 and iPIDD-2 were significantly less sensitive to etoposide-induced apoptotic cell death than E1A-positive control cells, whereas scRNA expressing E1A-positive cells remained equally susceptible (Figure 5b). The results in Figures 4a and b showed that caspase-2 expression is required for enhanced, etoposide-induced mitochondrial injury of E1A-positive cells. As was observed for caspase-2 shRNA-expressing cells (E1A-iC2), there was a marked reduction in the loss of MMP of iPIDD-1 cells treated with etoposide, when compared with E1A-positive control cells (Figure 5c).

One possible mechanism of E1A enhancement of caspase-2 activation in response to DNA damage could be increased basal expression of PIDD.^24^ However, full-length PIDD (PIDD-FL) expression was the same in E1A-positive and E1A-negative cells (Figure 5a). These results suggested that E1A might alter the activation state of PIDD, rather than its net expression.

### Cleavage of PIDD to PIDD-CC is required for enhanced cell death and caspase-2 activation in E1A-positive cells

**Figure 6:**
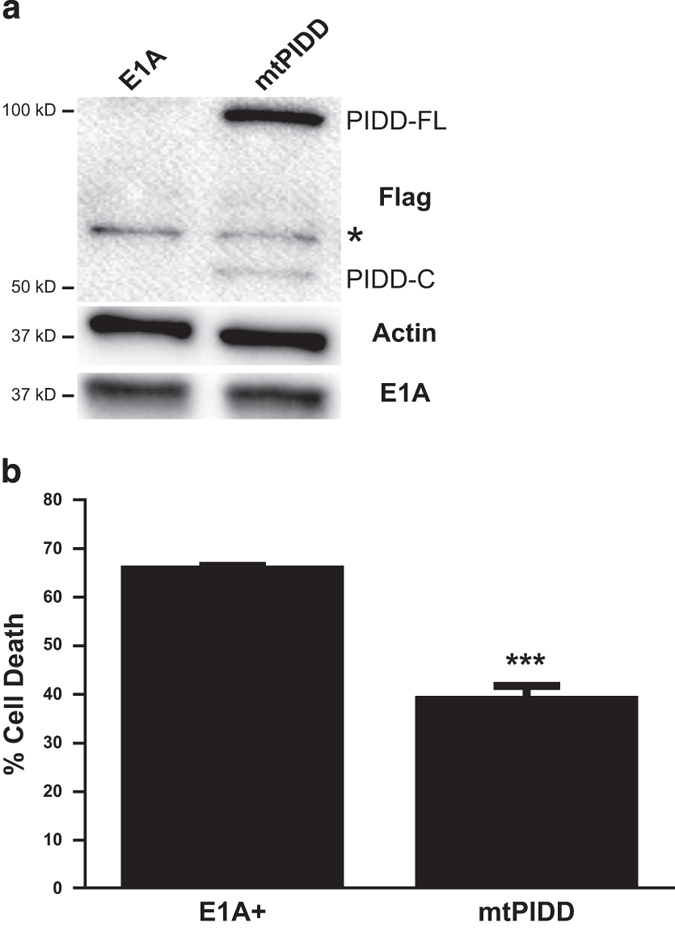
Requirement for PIDD cleavage to PIDD-CC for E1A-enhanced cellular sensitivity of mouse cells to etoposide-induced apoptosis. (**a**) Western blot for expression of Flag-tagged PIDD-S588A (mtPIDD) (M2), actin and E1A (M73) in E1A-positive control (E1A) and E1A+ mtPIDD cells. Full length (PIDD-FL) and PIDD-C are indicated. A nonspecific band is indicated by an asterisk. (**b**) E1A+ and E1A+ mtPIDD cells were treated with etoposide for 18 h. Cell viability was determined by MTS staining and expressed as % cell death (mean±S.E.M.; *n*=3, ***P<0.001).

The requirement of PIDD expression for E1A-enhanced apoptosis in response to DNA damaging agents suggested the importance of the PIDDosome for this E1A activity. PIDD must undergo two serial cleavage events to generate the caspase-2 activating form, PIDD-CC.^24^ To determine whether PIDD-CC was required for E1A-enhanced sensitization to DNA damaging agents, we created an E1A-positive mouse cell line (mtPIDD) that expressed c-terminal Flag-tagged PIDD-S588A, a mutant that cannot be cleaved to PIDD-CC and can act as a dominant negative mutant^27^ (Figure 6a). Overexpression of PIDD-S588A reduced etoposide-induced cell death of mtPIDD cells to a similar extent as observed with the E1A-positive iPIDD cells in which full-length PIDD expression was knocked down (Figure 6b compared with Figure 5b). These data indicated that PIDD processing to PIDD-CC is required for the enhanced chemosensitivity of E1A-positive cells, suggesting that the PIDDosome is a key caspase-2 activation platform required for E1A-induced sensitivity to apoptotic injury by these chemotherapeutic agents.

## Discussion

A wide variety of cellular pathways are targeted by E1A to increase cellular sensitivity to apoptosis.^33^ This diversity of effects raises the question of whether there might be a limited number of central, E1A control mechanisms that are amplified through secondary cellular networks. One such candidate for a central mediator of diverse E1A effects is the NK-*κ*B activation pathway, which participates in the control of over 150 cellular target genes, including several involved in the response to apoptotic stimuli.^44^ We and others have reported that E1A represses stimulus-induced NF-*κ*B-dependent cellular responses, including those that defend cells against apoptosis induced through extrinsic signals.^18,45,46^ We have also recently reported that there are NF-*κ*B-independent mechanisms through which E1A sensitizes cells to intrinsic apoptotic pathways triggered by macrophage-produced NO.^22^ The questions addressed in the current studies were whether E1A induced sensitivity to proapoptotic chemotherapeutic drugs is NF-*κ*B-dependent and whether this chemosensitizing activity of E1A is related to its enhancement of the upstream pathway of caspase activation, as we reported for E1A-induced cellular sensitivity to macrophage-produced NO.^22^ The results favor the latter hypothesis. Furthermore, the data indicate that E1A repression of the NF-*κ*B activation response and enhancement of stimulus-induced caspase-2 activation can be independent E1A activities.

Although our data indicate that optimal E1A-induced cellular chemosensitivity requires PIDD expression and caspase-2 activation, caspase-2 repression with either zVDVAD (Figures 3a and b) or siRNA (Figure 3c) did not eliminate E1A-related chemosensitization to the extent observed with the pan-caspase inhibitor, zVAD (Figure 1d). This partial repression of E1A-induced chemosensitivity was also observed with knockdown of upstream PIDD (Figure 5b) and expression of the dominant negative PIDD mutant, PIDD-S588A (Figure 6b), both of which reduce caspase-2 activation. We have observed such a lack of a complete blockade of the apoptotic response with NO-induced injury of E1A-expressing cells.^22^ One possible explanation is that the residual caspase-2 in inhibitor-treated cells is sufficient to mediate some level of apoptotic response. Another possibility is that a caspase(s), other than caspase-2, might also be activated in response to chemotherapy-induced DNA damage in E1A-positive cells. In any case, the data presented here clearly show that enhancement of the PIDD-caspase-2 cellular pathway is a major mechanism through which E1A increases cellular chemosensitivity.

Our data indicate that caspase-2 is the ‘apical’ caspase that mediates the cascade of mitochondrial injury and post-mitochondrial caspase activation events in chemotherapy-injured E1A-positive cells. This conclusion is supported by data with E1AiC2 (shRNA caspase-2) cells in which mitochondrial injury was blocked, as evidenced by reduction in injury-induced loss of MMP and release of cytochrome *c* into the cytosol (Figure 4). These results agree with reports of others, showing the requirement for caspase-2 to induce mitochondria-mediated apoptotic cell death in response to genotoxic chemotherapeutic agents.^38,47–49^ However, our data are the first to show that E1A enhances this ‘upstream’ caspase-2 activation in response to chemotherapeutic drugs. This finding is similar to our report that E1A enhances caspase-2 activation in response to macrophage-produced NO.^22^ We, therefore, propose that E1A sensitization to other intrinsic apoptotic injuries might proceed through this same enhanced caspase-2 activation response.

Caspase-2 can be activated through association with a high molecular weight complex called the PIDDosome.^24^ Caspase-2 activation in response to DNA damaging agents can be either PIDD-dependent or PIDD-independent.^27,30,31,50^ PIDD-independent caspase-2 activation might be the result of the effects of other macromolecular complexes, which, like the PIDDosome, can serve as platforms for procaspase-2 activation.^30,31^ The data presented here provide several lines of evidence indicating that the caspase-2-dependent apoptotic response of E1A-expressing cells to DNA damaging agents is dependent on the PIDDosome. First, repression of PIDD expression in E1A-positive cells resulted in a significant reduction in cell death induced by etoposide (Figure 5b). This reduction in chemosensitivity was similar to that seen with caspase-2 shRNA cells (E1AiC2; Figure 3c). Second, as shown in Figure 5c, PIDD-shRNA-expressing E1A-positive (EIA iPIDD-1) cells exhibited decreased mitochondrial injury in response to etoposide, as evidenced by measurement of MMP. Again, these results were similar to those with E1AiC2 cells (Figure 4a). Third, cleavage of PIDD from PIDD-C to PIDD-CC (required for PIDDosome formation) was required for enhanced chemosensitivity of E1A-positive cells (Figure 6b). Therefore, PIDD expression and processing to PIDD-CC is required for optimal E1A-enhancement of caspase-2-mediated apoptosis following DNA damage, confirming the importance of the PIDDosome for E1A-induced chemosensitivity.

**Figure 7:**
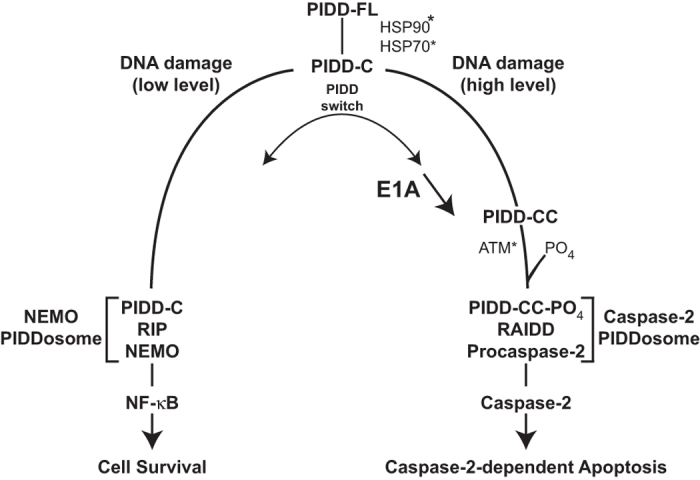
Model of E1A enhancement of PIDD-dependent Caspase-2 activation. It has been proposed that PIDD processing acts as a molecular switch that determines the cell fate following DNA damage.^27^ In cases of low level cellular DNA damage, PIDD might be preferentially processed to PIDD-C, favoring formation of the NEMO PIDDosome complex that activates NF-*κ*B, resulting in cell survival. In contrast, in circumstances resulting in high level DNA damage, as might be the case with E1A-expressing cells, PIDD might be preferentially processed to PIDD-CC, which leads to formation of the Caspase-2–PIDDosome complex, resulting in caspase-2-dependent apoptosis. E1A expression increases expression of HSP90 and HSP70 and activates the ATM signal transduction pathway (denoted by *) – all of which increase the PIDD activation cascade. E1A enhancement of these cellular activities might explain how E1A throws the cellular PIDD switch to favor Caspase-2–PIDDosome formation and caspase-2-related apoptosis.

PIDD has been proposed as a signaling protein that serves as a switch between cellular survival and death.^27,43^ The first-step cleavage of full-length PIDD (PIDD-FL) to PIDD-C that occurs at a low level of cellular DNA damage creates a PIDD molecule that activates NF-*κ*B-dependent cellular defenses against apoptosis, through the activity of the NEMO PIDDosome (Figure 7). The second step cleavage of PIDD-C to PIDD-CC that occurs at higher levels of cellular DNA damage pushes the balance away from PIDD-C-mediated NF-*κ*B activation and toward Caspase-2 PIDDosome formation that results in processing of procaspase-2 for caspase-2-dependent apoptosis. Our data indicate that E1A expression favors processing of PIDD to PIDD-CC, thereby pushing the balance away from the potential NF-*κ*B-dependent antiapoptotic defense and toward increased caspase-2-dependent apoptosis – essentially throwing the cellular ‘PIDD switch’ to enhance the cell death response to chemotherapeutic drug injury (Figure 7).

The molecular mechanisms through which E1A enhances PIDD-dependent activation of caspase-2 remain to be defined. However, there are several points in the caspase-2 activation pathway where E1A might affect PIDD function (Figure 7, arrowheads). One potential target is regulation of PIDDosome formation. The caspase-2 PIDDosome is composed of phosphorylated PIDD-CC, procaspase-2 and RAIDD.^24,25^ Formation of the PIDDosome, autoprotolytic processing of PIDD and PIDD-CC phosphorylation are key steps in recruiting procaspase-2 to the complex and enabling caspase-2 activation by the induced proximity model.^26,27,51^ Biochemical studies have shown the rate-limiting step in the formation of the PIDDosome to be the interaction of PIDD-CC and RAIDD.^52^ One mechanism through which E1A could alter PIDDosome formation could be increased basal PIDD expression, resulting in an increased PIDDosome platform. However, no such E1A-related increase in basal PIDD expression was observed in our studies (Figure 5a). The alternative possibility would be E1A enhancement of the processing of basal PIDD. Hsp90 and Hsp70 are chaperones that stabilize PIDD and that are involved in processing of PIDD-FL to PIDD-C and of PIDD-C to PIDD-CC.^27^ Expression of E1A increases expression of both Hsp90 and Hsp70.^53,54^ Therefore, it is possible that E1A-induced Hsp90 and Hsp70 expression enhances the PIDD activation cascade. Another possible mechanism could involve E1A enhancement of phosphorylation-induced activation of PIDD. Sidi *et al.* have shown that signal transduction through the DNA damage response pathway plays a key role in regulating caspase-2 activation through phosphorylation of PIDD by ATM, the protein kinase product of the ataxia-telangiectasia mutated gene.^51,55^ ATM expression is induced by DNA damage and is also induced, independently of DNA damage, by E1A, through an Rb-E2F transactivation mechanism, to promote apoptosis.^56^ It is therefore possible that E1A might enhance ATM-dependent PIDD phosphorylation, resulting in enhanced PIDDosome formation following chemotherapy drug-induced DNA damage. In addition to all of these potential modification steps in the PIDD-dependent caspase-2 activation pathway, E1A expression results in a general increase in the cellular DNA damage response, by direct effects of E1A on cellular double-stranded DNA breaks and by its indirect effects through E2F activation and through E1A repression of PARP, which initiates several different DNA repair mechanisms.^57,58^ These potential multifactorial mechanisms of an E1A increased cellular DNA damage response would be predicted to increase the cellular PIDD-CC to PIDD-C ratio, further favoring a switch away from PIDD-C-induced NF-*κ*B-dependent antiapoptotic defenses and toward PIDD-CC-induced, caspase-2-dependent cellular death.^27^

In summary, the results show that E1A-mediated sensitization to the DNA damaging, chemotherapeutic agents, etoposide and gemcitabine, require the expression of caspase-2. Caspase-2 is the apical caspase activated in response to DNA damage in E1A-expressing cells and is required for drug-induced mitochondrial injury. Furthermore, the caspase-2-induced mitochondrial injury requires both the expression of PIDD and its autoprocessing to PIDD-CC for efficient cell death to occur in response to etoposide. We speculate that some of the functions of E1A that have evolved to increase viral replication in quiescent mammalian cells have collateral effects that enhance the PIDD-dependent caspase-2 activation pathway, resulting in increased cellular chemosensitivity. Further definition of these E1A mechanisms could have implications for enhancement of tumor cell sensitivity to chemotherapeutic agents during adenovirus-based virotherapy.

## Materials and Methods

### Cell lines

NIH-3T3 cells expressing Ad5 E1A 12S (MT12-1) or 13S (13-2) and human H4 cells (a subclone of the human fibrosarcoma cell line, HT1080) expressing genomic E1A (H4-E1A, P2AHT2A) and their derivatives were maintained at 37 °C and 5% CO_2_ in DMEM plus antibiotics and 5% calf serum.^11,34^ The TNF-resistant (E1A+ TNFr) and p65/RelA-overexpressing (E1A+ p65) NIH-3T3 E1A 12S cell lines and an NIH-3T3 E1A 12S line expressing an shRNA targeting caspase-2 (E1AiC2) have been described.^22^ All cells lines were negative for mycoplasma contamination.

### PIDD shRNA E1A-positive cells

NIH-3T3 E1A 12S cells were infected with lentiviral particles containing shRNA for mouse PIDD or a scrambled target sequence (SC), along with a puromycin resistance marker and a GFP gene (Dharmacon, Lafayette, CO, USA). Following selection in 8 *μ*g/ml puromycin, single-cell clones were screened for GFP expression, and high GFP expressing cell lines were screened for sustained expression of E1A (anti-E1A M73 antibody), actin (Sigma, St Louis, MO, USA) and repressed expression of PIDD (LISE-1; AdpioGen, San Diego, CA, USA) by western blot. The knockdown cell lines, iPDD-1 and iPIDD-2, were generated with the following siRNA target sequence: AGCTTTAAACTTGACTCGA.

### PIDD S588A E1A-positive cells

A plasmid encoding C-terminal Flag-tagged PIDD-S588A was obtained from the Jürg Tschopp Laboratory.^27^ Flag-PIDD-S588A was cloned into pcDNA3.1 (+) Hygro (Invitrogen, Carlsbad, CA, USA) by *Hin*dIII/*Not*I digestion and ligation. NIH-3T3 E1A 12S cells were electroporated with the resulting pcDNA-PIDD-S588A vector and selected in 200 *μ*g/ml hygromycin. The resulting clonal cell line, mtPIDD, was screened for expression of E1A and Flag-tagged PIDD-S588A (anti-Flag M2; Sigma) by western blot.

### Cytotoxicity assays

Etoposide and gemcitabine induced cytotoxicity was measured by radiolabel release from target cells as described.^59^ Briefly, cells were labeled with Cr^51^ for 1 h at 37 °C and washed two times to remove free Cr^51^. Labeled cells were incubated in the indicated drug concentrations for 18 h, after which Cr^51^ released into media was quantitated by liquid scintillation counting. For experiments using the pan-caspase inhibitor zVAD (100 *μ*M; Calbiochem, San Diego, CA, USA) or the caspase-2 inhibitor zVDVAD (200 *μ*M; Calbiochem), radiolabeled cells were incubated with the inhibitors for 2 h prior to addition of drugs. In some experiments, a non-radiolabel cytotoxicity assay was also used (MTS; Promega, Madison, WI, USA). The MTS assay results were identical to those obtained using Cr^51^ release assays.

### Evaluation of cell death phenotype

Cells were treated with either etoposide or gemcitabine, as described in the figure legends. At the indicated end points, floating and adherent cells were collected, washed two times in PBS and resuspended in 1 ml of PBS+1% FBS. Cells were stained with Hoechst and propidium iodide (PI) (Vybrant Assay 5; Invitrogen) prior to data acquisition by flow cytometry.

### Assessment of MMP

Cells were treated with either etoposide or gemcitabine as indicated. Floating and adherent cells were collected and processed following treatment as described above. Cells were then stained with 100 nM tetramethylrhodamine ethyl ester perchlorate (TMRE; Invitrogen) or with 25 nM MitoProbe DilC_1_ (5) (DilC; Invitrogen) for 30 min at 37 °C prior to data acquisition by flow cytometry.

### Flow cytometry and data processing

Data acquisition was performed on either BD LSR (BD Biosciences, San Jose, CA, USA) for Hoechst/PI and TMRE or Accuri C6 (BD Biosciences) for DilC. Flow data were processed using FlowJo X 10.0.7r2 (Tree Star Inc., Ashland, OR, USA). Cells were analyzed first by forward scatter *versus* side scatter, and a gate was established to exclude cellular debris from comparative analysis using untreated control cells. This gate was then applied to all data from the same acquisition. A quadrant was set to assess Hoecsht/PI staining, and the same quadrant was applied to all data from the same acquisition set. The same non-debris gate was used to generate histograms of TMRE or DilC.

### Cytochrome *c* release

Cells treated with etoposide or gemcitabine for 4 h were washed twice, and cytosolic and mitochondrial fractions were isolated, using Mitochondria Isolation Kit (Pierce, Rockford, IL, USA). Cytosolic fractions were probed for cytochrome *c* and Cox IV (a mitochondrial marker) with antibodies from the ApoAlert Cell Fractionation kit (Clontech, Mountain View, CA, USA), using actin (Sigma-Aldrich) as a loading control.

### Caspase activity assays

Caspase-2 and caspase-3 activity was assessed as follows. Cells were treated as described for 4 h at 37 °C and then washed two times with cold PBS and resuspended in 50 *μ*l of cell lysis buffer and incubated on ice for 10 min. Clarified cell lysates were used to determine caspase activity with either Caspase-2 or Caspase-3 Activity Assay (R&D Systems, Minneapolis, MN, USA). Activity was expressed as the percentage of untreated control cell activity.

### Statistical analysis

Statistical analysis was done using Student’s two-tailed *t-*test, with SigmaPlot 12.3 software (Systat Software, San Jose, CA, USA). Normal distribution was confirmed by Shapiro–Wilk. Data are expressed as mean±S.E.M. of at least three independent experiments.

## Abbreviations

E1A: Adenovirus early region 1A gene
E1A-iC2: E1A positive caspase-2 siRNA cells
iPIDD: E1A positive PIDD siRNA cells
MMP: mitochondrial membrane potential
mt-PIDD: PIDD with a mutation of Serine 588 to Alanine
NF-κB: nuclear factor kappa B
NO: nitric oxide
PIDD: p53 inducible death domain containing protein
PIDD-C: first carboxy terminal PIDD cleavage product
PIDD-CC: carboxy terminal product following cleavage of PIDD-C
PIDD-FL: full length PIDD
RAIDD: RIP-associated death domain containing protein
scRNA: scrambled shRNA
TMRE: Tetramethylrhodamine ethyl ester perchlorate
